# Aesthetic Gynaecology: What Women Want?

**DOI:** 10.7759/cureus.44251

**Published:** 2023-08-28

**Authors:** Shazia Mohammad, Ketav S Joshi, Shirin Mohammad, Neema Acharya

**Affiliations:** 1 Obstetrics and Gynaecology, Jawaharlal Nehru Medical College, Datta Meghe Institute of Higher Education & Research, Wardha, IND; 2 Otolaryngology - Head and Neck Surgery, NKP Salve Institute of Medical Sciences, Nagpur, IND

**Keywords:** female cosmetic gynaecology surgery, radiofrequency, rejuvenation, vaginoplasty, labiaplasty, aesthetic gynecology

## Abstract

The demand for aesthetic gynecology is growing among patients and medical professionals. It is becoming a field of increasing interest. In obstetrics and gynecology, there are currently few superspecialization or fellowship training programs that teach this subject; nevertheless, improvements have been made in aesthetic and plastic surgery training that foresee the need to add specialized training in this sector. In the US and the UK, numerous reputable certification and preceptorship programs are now where many surgeons start their careers. To give physicians interested in surgical and non-surgical therapies certification training, new programs were introduced globally in 2016-2017. We provide an overview of both surgical and non-surgical "aesthetic gynecology" treatments, as well as the opinions of the top gynecologic associations on this new field of study.

## Introduction and background

The changing views of beauty are influenced by advancements in technology and fashion. Furthermore, social and cultural norms differ across countries, adding to the variation. Due to these differences, it is challenging to represent the typical view of female external genitalia accurately. According to Hodgkinson et al. [1], labia minora, which is smaller, and labia major, which is larger in proportion, are deemed aesthetically ideal.

Motakef’s classification system is based on the degree of protrusion of the labia minus compared to the labia majus [2]. Similarly, Hamori et al. classified labias based on their morphological variations and shapes [3]. However, these classification systems are not relied upon by gynecological or plastics societies, nor are they used by general practitioners. Labiaplasty is required for surgical intervention in labial hypertrophy and congenital adrenal hyperplasia (CAH) cases. Nonetheless, most patients who have labiaplasty do so to reduce discomfort when exercising or wearing clothing due to saggy skin or for uninterrupted sexual experience.

Individuals may decide to have genital surgery if they are self-conscious about the way their genitalia look [4]. These concerns may include the perception of size as the larger or asymmetrical appearance of the labia minora and a darker appearance of the labia majora. Determining an ideal standard for genital appearance is challenging and should be individualized.

Aesthetic gynecology has experienced a surge in popularity in recent years due to its positive impact on health and appearance. Modern women are increasingly mindful of their bodies and are embracing innovative medical procedures to attain their desired aesthetic goals, whether they pertain to their external appearance or intimate areas. It is important to note that aesthetic gynecology extends beyond sexual enhancement and significantly promotes women's reproductive health and functionality. Additionally, one's emotional well-being is directly influenced by body perception. Individuals who embrace and accept their bodies tend to exhibit higher confidence and happiness.

## Review

Aesthetic gynecology surgical techniques

Aesthetic gynecology involves various procedures, including labiaplasty, clitoral hood reduction, and vaginal rejuvenation, which includes vaginoplasty and perineoplasty.

Labiaplasty

Labiaplasty, also called labioplasty, is a commonly requested cosmetic gynecological surgical procedure. It aims to surgically alter and frequently reduce the size of the labia majus or minus while preserving a consistent lip contour and labial edge color to reduce obstructing tissues during sexual intercourse. Hodgkinson and Hait first discussed this procedure in literature [1]. A variety of surgical techniques, including linear incision, de-epithelialized reduction, composite reduction, curvilinear resection, V-wedge resection, wedge reduction, inferior wedge resection, W-plasty excision or Z-plasty, superior pedicle flap reconstruction, and other less common procedures, are available. However, preserving the natural contour of the corrugated free edge with these techniques is difficult. The procedure must be tailored to the individual patient's needs. In a study of 550 women, Magon et al. in 2017 [5] found that 97% of them requested the removal of dark edges and had labia that were flush with or tucked beneath the labia majus, with smaller and straighter labia with a pinker edge, which is considered advantageous. The linear excision technique is commonly used among gynecologists due to its simplicity and minimally invasive nature. In some areas, such as Laguna Beach, California, terms such as "rim look," "Barbie look," or "hybrid look" may be used to describe labial reductions based on the level of the labia majora in proportion to minora [6].

Labia majora plasty is a surgical option that can treat medical conditions such as congenital lymphedema and chronic steroid-induced sagging associated with steroid use for CAH. When done for aesthetic reasons, labia minora plasty and labia majora plasty are typically performed simultaneously, although they are distinct procedures.

Clitoral Hood Reduction

The clitoral hood, known as the preputium clitoridis, is a flap of skin covering the external portion of the clitoral glans, also called the clitoral head. Clitoral hood reduction, or clitoral hoodectomy, is a voluntary surgical intervention that removes excess tissue from the fold surrounding the clitoral glans while separating the prepuce from the clitoral tissue. According to Goodman (2009) [7], it is crucial to differentiate this operation from clitoridectomy.

Patients may seek a clitoral hood reduction to increase sexual satisfaction by exposing more of the clitoris or for aesthetic, hygienic, or comfort reasons, such as alleviating chafing or discomfort caused by a trapped clitoris [8]. Surgery serves to reduce the clitoral prepuce's length and prominence surgically. According to Kent et al.'s (2012) study of 407 patients who underwent clitoral hood reduction and central wedge labiaplasty, the procedure has a low complication rate when performed by skilled surgeons, with a revision surgery rate of only 2.9% [8].

Vaginal Rejuvenation

Vaginal rejuvenation is a surgical procedure that combines perineoplasty and vaginoplasty to address a widened vaginal canal. These techniques are variations of well-established procedures, such as pelvic floor reconstruction and colpoperineorrhaphy [9].

Vaginoplasty, another name for vaginal tightening, is a surgical technique that entails changing the epithelium, deeper canal, and vaginal entrance. Contrary to popular belief, pelvic floor repair should be examined as part of a urogynecologic evaluation. To address concerns about a big or loose vagina, the operation may involve the lateral vaginal mucosa excision or high posterior repair. It is frequently performed with perineoplasty and paravaginal repair, with or without an anterior colporrhaphy. Communication between surgeon and patient is important to determine the extent of vaginal diameter reduction that is desired and feasible and to manage patient expectations. The risks of over-tightening should be discussed with the patient [10].

**Figure 1:**
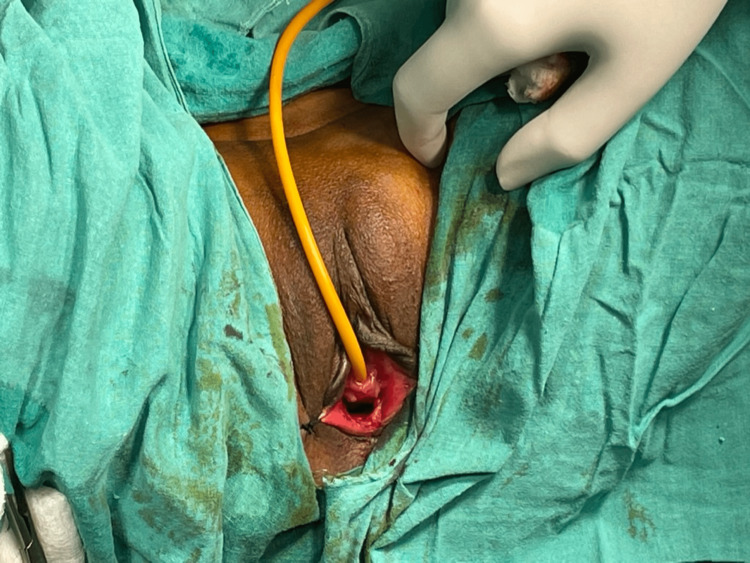
Vaginoplasty The picture is the authors' own creation.

One technique used in vaginoplasty involves removing excessive vaginal mucosa from the fornices (Figure 1), while another option is to perform excision of lateral vaginal mucosa or anterior/posterior colporrhaphy or a combination of these methods to achieve the desired outcome.

Perineoplasty, or perineorrhaphy, is a surgical procedure aimed at reconstructing the vaginal introitus by reducing the size of the vaginal opening and tightening the perineal muscles. It is often performed in combination with posterior colporrhaphy, which is the surgical repair of a weakened or damaged vaginal wall in cases of pelvic organ prolapse [11].

The primary objectives of perineoplasty surgery are to improve the appearance of the perineal area and enhance sexual satisfaction by lifting the perineum and increasing friction during penile penetration. Additionally, it can improve the mechanics of defecation by correcting the path that stool passes through. Perineoplasty, if performed alone to address sexual dysfunction, can be ineffective [12].

Reverse perineoplasty is another procedure variation that involves reconstructing scar tissue from previous surgery or lichen sclerosus, a skin condition that affects the vulva. The procedure involves removing visible bands and scars and building an advancement flap to improve the introital caliber [13].

Labia Majora Augmentation

Labia majora augmentation is a nonsurgical procedure that aims at improving the aesthetic appearance of labia majora that are hypoplastic or loose. Hyaluronic acid (HA) fillers and autologous fat grafting are common procedures. Autologous fat grafting can be sourced from various fatty areas, but typically from the thigh or inner knee, and is prepared using techniques such as the Coleman method [14]. However, re-absorption of the graft must be considered to achieve the desired outcome. HA fillers are more popular in Europe due to their lower cost, while fat grafting is more common in the United States. In Europe, HA fillers are often used to enhance the labia majora, while in the US, labia majora-plasty or radiofrequency shrinkage is preferred for a sleeker appearance. The surgeon's skill and anatomical knowledge are essential for a successful outcome. Nonetheless, caution must be exercised when using HA in this region to prevent complications such as granuloma formation.

Hymenoplasty

In Western nations, hymenoplasty is frequently referred to as revirgination and involves several ethical concerns. The least researched female genital surgery is one that is mostly sociocultural. Several ethics committees do not view hymenoplasty as cosmetic genital surgery, and it is more reasonable to categorize it as a reconstructive rather than an aesthetic technique. The surgery may have significant cultural and social repercussions, yet it may also save some women's lives. To safeguard their privacy, patients can be told that the procedure is a "vaginal repair," a reasonably straightforward surgery [15].

Aesthetic gynecology nonsurgical techniques

LASER Treatment for Vaginal Laxity

**Figure 2:**
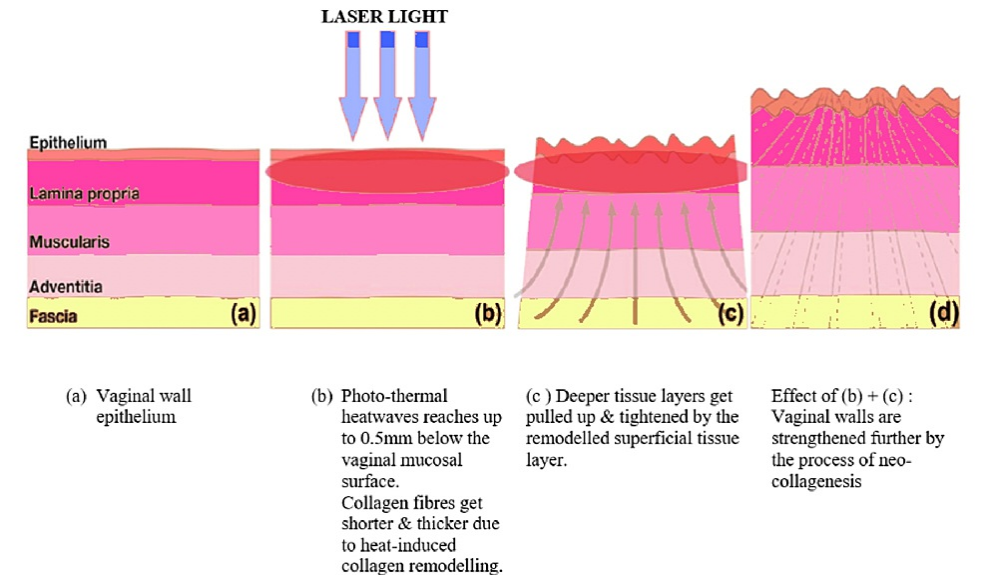
Mechanism of action of LASER for vaginal rejuvenation The author has self-created the figure.

Vaginal laxity can now be treated without surgery using fractional lasers. The fractional carbon dioxide (CO2) laser, which has a wavelength of 10.600 nm, is one of them and is useful because it can absorb water from tissues, which causes the vaginal mucosa to hydrate, regenerate collagen fibers, and regain its suppleness (Figure 2). On the other hand, the fractional erbium laser has a wavelength of 2,940 nm and has been applied to treat stress incontinence, vaginal tightness, and postmenopausal vulvar-vaginal atrophy. Collagen fibers contract under the influence of the erbium laser, which has a water absorption affinity roughly 10-15 times greater than the fractional CO2 laser. This causes tissue shrinking. Moreover, the fractional erbium laser reduces heat damage to the tissue around it, reducing postoperative discomfort and edema [16].

Matlock, who used a 980-nm diode laser as a cutting tool comparable to traditional cautery during the surgery, first registered the term "laser vaginal rejuvenation" as a trademark [17]. However, many laser companies now use newer, minimally invasive fractional laser techniques to shrink the vaginal walls in what is often referred to as vaginal laser therapy or laser vaginal resurfacing. It is important to note that there is some controversy surrounding the use of laser vaginal rejuvenation, as some medical professionals have raised concerns about the safety and effectiveness of the procedure [17].

*Radiofrequency *(*RF) Vaginal Rejuvenation*

Using energy-based skin rejuvenation techniques, vulvovaginal laxity brought on by aging or childbearing has been treated. Studies show that using RF to repair vaginal tissue boosted collagen and elastin production [18]. In naturally moist tissue, RF outperforms laser therapies, which are skin-type dependent. Temperature-controlled RF, which utilizes thermistors for temperature monitoring and thermostating at the target tissue temperature of 40-45 degrees Celsius, shows a promising safety profile.

This technique helps promote healthy tissue formation by facilitating collagen denaturation and healing, resulting in tightening. The immediate tightening observed after the procedure is thought to be due to the production of thicker, shorter collagen fibers that occur when the tissue is heated.

Because RF technology can encourage the production of new elastin [19], which is uncommon in other procedures, it may be beneficial for treating vaginal laxity. Increased local blood flow from RF can also improve sexual performance and satisfaction by reducing dryness caused by vulvovaginal atrophy. Moreover, studies have indicated that RF can cause the pubocervical fascia to tighten and the stress urine incontinence to revert [20,21].

Vulvar Lightening

The use of chemical agents or fractional carbon dioxide laser method can help to lighten the hyperpigmented appearance of the vulva. However, preventing rebound hypopigmentation should be a primary goal. Energy-based tools such as CO2 lasers can lead to hyper- or hypo-pigmentation. Ablative use of RF can also result in pigmentation issues, but non-ablative RF can help to avoid these problems.

Platelet-Rich Plasma (PRP)

**Figure 3:**
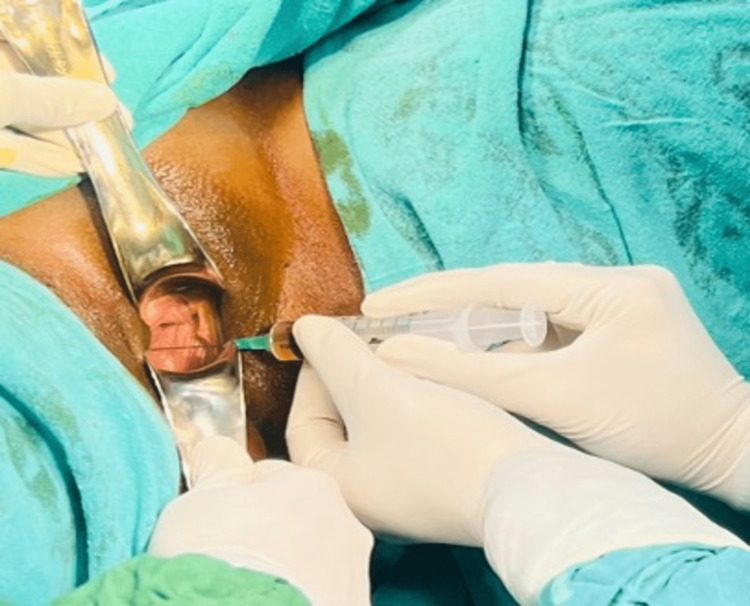
Autologous platelet-rich plasma injection in the vagina This picture is the authors' own creation.

Since it was initially published in 1987 for open-heart surgery, autologous PRP has undergone extensive research in several fields, including orthopedics, dentistry, wound care, and cosmetic surgical operations [22]. PRP contains large growth factors such as platelet-derived growth factor, transforming growth factor beta, and epidermal growth factor (Figure 3 showing PRP injection in the vagina). Because it is autologous, PRP is nonantigenic and usually has no negative effects. In addition to its surgical applications, PRP injections can potentially be nonsurgical treatments for female sexual dysfunction, poor lubrication, and stress urinary incontinence [23].

The amalgamation of PRP and RF has been identified as a promising approach for providing long-term symptomatic relief in lichen sclerosis, and ongoing studies are being conducted to investigate this treatment. The identification of an erotic zone along the anterior vaginal wall, along the urethral course, was first reported by Grafenberg in 1950 [24], and subsequent research has confirmed its existence. This region has been designated as the G-spot by Addiego et al. [25]. While its precise anatomy is not yet fully understood, it is thought to be a neurovascular complex associated with vaginally activated orgasm [26,27]. The augmentation of the G-spot region using fillers, such as collagen or autologous fat transfer, has been reported to increase the bulk of the vaginal lumen, potentially enhancing sexual activity [28].

HA Filler

A clinical diagnostic criterion was established by Ostrzenski [27] for a secondary dysfunction of the G-spot: (i) secondary difficulty in achieving orgasm through vaginal stimulation, (ii) reduced engorgement of the vagina, (iii) diminished sensation in the anterior-distal vaginal wall during sexual arousal, (iv) history of difficult vaginal birth or surgery of anterior distal vaginal wall, and (v) previous history of noninvasive medical treatment.

G-spot amplification, or G-spot augmentation or the G-shot, is a minimally invasive cosmetic surgical procedure that aims to enhance the size of the G-spot temporarily. The G-spot is believed to be located roughly halfway between the pubic bone and the cervix. The amplification process involves using HA filler, although, in some cases, autologous fat transplant is also utilized and injected into the bladder-vaginal septum. The basic idea behind the treatment is that expanding the G-spot has the potential to improve sensory input and friction, which could then heighten sexual enjoyment. Fillers made of HA have also been used to improve deflated or atrophic both the major and minor labia.

Lipofilling

Adipose-derived stem cells (ADCs) are a rich source of stem cells that are widely distributed. Autologous fat transfer carries a very low chance of rejection. ADCs are a component of a complex mixture called the stromal vascular fraction (SVF), which also includes endothelial cells, extracellular matrix, and different immune cells.

To increase the thickness of the vaginal walls, rejuvenate the skin of the vulva, and restore genital volume, lipofilling, a technique involving the transfer of fat, is carried out. By grafting fat into the labial folds, this procedure thickens them, thereby shrinking the vaginal diameter [29].

Requests for vulvovaginal cosmetic procedures in adolescents

For various reasons, whether to enhance their appearance, fix functional issues, or psychological ones, women may decide to undergo cosmetic treatments. Young girls and teenagers who choose to get labioplasty may have different motivations. To treat issues such as rubbing, chaffing, or disruption of sports, girls between nine and 13 may request surgery. Sometimes a mother will notice a problem with her daughter and contact a doctor. Young adults between the ages of 14 and 17 may primarily seek labioplasty due to concerns about looks and worries that they would not be viewed favorably by sexual partners [30].

Beyond the usual preoperative planning and counseling, additional considerations must be made when considering labioplasty for teenagers. These include the reason for the operation, any specific anatomical issues, the patient's physical maturity, societal costs, the dynamics of parent-child decision-making, and the patient's prognosis following the operation. It is crucial to consider the typical physiological and developmental changes in young females, especially in the vulva, and to delay treatments until the patient has reached a stage of mature genital development.

Advertising

It is significant to highlight that marketing cosmetic procedures such as labioplasty and vaginal rejuvenation can be contentious and create ethical questions. The advertisement might not accurately portray the operations' risks and probable side effects, which could lead to patients having inflated expectations. Additionally, the financial incentives of the medical professionals doing these procedures may cloud their judgment and result in pointless operations.

Gynecologists should put their patient's health and well-being before financial gain and ensure they are properly educated about the advantages and hazards of every surgery. While discussing procedures and potential benefits is acceptable, it is unethical to advertise them in a way that is misleading or promotes unnecessary surgery. Gynecologists should exercise professional discretion and respect ethical standards while deciding whether to advertise aesthetic procedures. They should also be honest about any conflicts of interest and always prioritize the safety and wellness of their patients.

Recommendations

Practice recommendations are mentioned in Table 1.

**Table 1:** Recommendations

Sr. No,	Recommendations
1.	Obstetricians and gynecologists need to educate women about their anatomy and that differences in appearance are normal and should be celebrated.
2.	Before performing any vaginal cosmetic procedure, a complete medical, sexual, and gynecological history should be obtained to ensure that the patient has no significant psychological or sexual disorders and is not being coerced or exploited.
3.	Women seeking genital cosmetic surgery should undergo counseling to discuss the potential risks and unintended consequences, as well as the normal variations and changes that occur with age, pregnancy, and menopause. Informed consent should consider the lack of information regarding long-term effects and results.
4.	Doctors need more training in adolescent counseling when they see teenagers asking for female genital cosmetic surgery. Parental consent is unnecessary at that point, and such procedures should not be offered until a person is fully mature, including their genitalia.
5.	There is limited evidence to support the idea that genital cosmetic surgery can improve sexual satisfaction or self-image. Therefore, advertising for these procedures should be avoided, and doctors who choose to perform them should not promote them as a way to improve sexual function.
6.	Female genital cosmetic surgery (FGCS) should be performed only after specialized training.
7.	They should also adhere to ethical guidelines and prioritize the patient's well-being over financial gain.
8.	Adolescent girls seeking vaginal cosmetic procedures should be evaluated on a case-by-case basis, with special consideration given to their age, anatomical concerns, physical maturity, and psychological well-being.
9.	Women should be informed about nonsurgical alternatives that may address their concerns, such as pelvic floor physiotherapy and counseling.
10.	There should be a super specialty training course for a branch of cosmetic gynecology.

## Conclusions

Despite a lack of evidence supporting the use of cosmetic surgery for female genitalia, the increasing popularity of these procedures is extremely alarming. Educating and counseling women on their anatomy, including the normal variances and changes that occur over time, is crucial to addressing this issue. To ensure that women are completely informed of the potential dangers and unintended consequences of genital cosmetic surgery, counseling should come before any informed consent procedure. Technology has advanced significantly over the past 10 years, providing new tools and methods for reversing the effects of aging on tissue remodeling and restoring tissue function. These developments have opened the door to investigating cosmetic uses in conventional gynecology, especially vaginal rejuvenation. It has been overdue for these practices to be socially accepted and for regulatory organizations to be set up to ensure their correct application. However, this discipline has effectively carved out its specialized area and has shown significant potential and promise in enhancing patients' quality of life. Cosmetic gynecology procedures address issues often considered taboo and not openly discussed. Ironically, women may not even bring up their symptoms with their doctors, partly due to a lack of awareness about such procedures and a limited acceptance of them to address functional problems or enhance self-esteem by improving aesthetic appearance. As this field continues to evolve rapidly, it becomes crucial to establish standardized procedures for safer practice. Long-term studies are necessary to understand the outcomes of these procedures and validate the efficacy of novel approaches within this field.
